# Development of a Clinically Relevant Index for Tooth Wear Treatment Needs

**DOI:** 10.3390/dj10050080

**Published:** 2022-05-09

**Authors:** Yahya Deeban, Keyvan Moharamzadeh, Moosa Abuzayeda, Nicolas Martin

**Affiliations:** 1 School of Clinical Dentistry, University of Sheffield, Sheffield S10 2TA, UK; yahyadeeban@gmail.com (Y.D.); n.martin@sheffield.ac.uk (N.M.); 2 Department of Prosthodontics, College of Dentistry, Majmaah University, Al-Majmaah 11952, Saudi Arabia; 3 Hamdan Bin Mohammed College of Dental Medicine (HBMCDM), Mohammed Bin Rashid University of Medicine and Health Sciences (MBRU), Dubai P.O. Box 505055, United Arab Emirates; moosa.abuzayda@mbru.ac.ae

**Keywords:** tooth wear index, tooth surface loss, aesthetics, tooth wear functional impact

## Abstract

Background: This study aimed to develop a tooth wear classification system that combined the extent, severity, and aesthetic impact of tooth wear and correlated them with the most appropriate clinical management strategy. Methods: Three hundred photographs were used to develop a classification tool that contained four levels of severity and aesthetic impact (0, 1, 2, and 3) in three age groups of patients. Ten examiners assessed and classified the cases using validated forms. Additionally, they selected the recommended treatment modality for each level. The analysis was conducted using a coefficient correlation test. Results: The coefficient correlation for the severity was 0.81, 0.82 in the upper anterior and posterior segments, and 0.85 and 0.77 for the lower anterior and posterior segments, respectively. The aesthetic impact correlation coefficient was 0.84. Examiners had agreed that minor cases required monitoring or simple restorative interventions. The moderate-level cases had variety in the recommended management options depending on the aim of treatment. The severe level cases often required rehabilitation at an increased occlusal vertical dimension. Conclusion: Within the limitations of this preliminary study, a good agreement between the examiners was found using the provided tools. More strict criteria in the classification part of the tool can further improve the examiners’ agreement.

## 1. Introduction

Tooth surface loss (TSL) is an increasingly common and destructive pathology that lacks rigorous criteria to assess the disease severity and treatment needs, even though numerous attempts have been made to develop wear indices and classification systems [1]. The high incidence of different clinical manifestations of TSL is a growing dental problem, and its severity can increase with patients’ age [2]. Moreover, it has been found that about 30% of European young adults, aged 18–35, suffer from TSL [3]. The worn dentition can cause functional impairments, e.g., tooth sensitivity, especially in cervical lesions [4,5,6,7]. Regarding aesthetics, Katsoulis et al. (2011) found the majority of patients with worn dentitions attending dental clinics have their key complaint about the aesthetic impairment of anterior teeth [8]. Poor appearance from severe TSL can be associated with low self-awareness, increasing self-consciousness, and discomfort in daily jobs [4]. For these reasons, an index that considers the impacts of aesthetic parameters in addition to severity on the treatment strategy would appear to be a crucial element during assessment of TSL patients.

Many studies have attempted to provide classification systems for the different TSL manifestations (Table 1). However, many of these are of academic interest with a focus on classification for research purposes or considered too complex to implement into routine primary dental care, such as the Eccles classifications in 1978 and 1979 [9,10]. Moreover, there is no universal and validated index combining the functional and aesthetic elements of TSL that can be used to recommend case-specific and relevant treatment options.

In 1984, Smith and Knight introduced a tooth wear index (TWI). This index was more practically and clinically relevant compared to previous indices [9]. However, it is difficult, in practice, to measure all surfaces of each tooth. In addition, giving the lowest score measured for each tooth, according to the given criteria, can lead to underestimation of the intervention required [11]. Bardsley et al. (2004) developed a simplified version of the TWI [12]. This index is straightforward to adopt clinically compared to measuring all surfaces in the initial TWI by Smith and Knight (1984). However, it can give an underestimation of TSL incidence in patients with enamel TSL, who were scored with a 0 index. This can then lead to more progression of enamel lesion, which would be arrested by a preventive approach if it could be diagnosed early.

The Basic Erosive Wear Examination (BEWE) was introduced by Bartlett et al. (2008). It aimed to provide easy and reproducible diagnostic criteria to evaluate and measure the severity of TSL, then assist in the management decision required [13]. The reliability of BEWE was assessed and compared to TWI by Bartlett (2012). The results showed high sensitivity and specificity in scores 3 and 4 only [14]. However, detection of early loss of tooth surface is much more important from the clinical perspective to prevent the progression of the lesion and be able to undertake conservative management without applying excessive intervention.

The new tooth wear index (NTWI) was recently published by Soo-Hyun Kim et al. (2018) [15]. This index evaluated quantitative parameters using the cusp area. The parameters assessed distances and angles from the cusp tip. However, this index requires a clear occlusal plane and is not suitable to quantify the TSL in severe cases where the occlusal plane is not suitable.

This preliminary investigation seeks to develop an ‘Index for Tooth Wear Treatment Need (IWTN) ’using clinical photographs. This classification will apply similar principles to the universally accepted index of orthodontic treatment needs (IOTN) but with TSL features and clinical manifestations. IOTN was developed by Brook and Shaw (1989) [16] and used a series of photographs describing all relevant conditions and the priority for treatment for each level. To develop a photographic classification for tooth surface loss similar to IOTN, it is important first to find an easy and reproducible tool to categorize the TSL clinical photographs into different severity and aesthetic impact levels and correlate each level to the suitable management modalities.

The hypothesis is that using clinical photographs can provide a predictable tool to classify the severity and aesthetic impact of TSL, and it can provide a suitable management approach. This preliminary investigation aims to develop and test a classification tool to categorize clinical cases of TSL and their correlation to the recommended treatment modalities, considering the aesthetic impact and severity of the disease. This tool and supporting photographic version could help dentists clinically assess and provide management options for different manifestations of TSL. The study question was: can we establish a clinically relevant and easy-to-use index of treatment needs for the management of TSL?

**Table 1:** TSL indices and classification systems.

Year	Author	Aim of the Index
Erosion classification systems		
1978	Eccles	Early classification of erosion TSL of industrial origin [10].
1979	Eccles	Modified erosional TSL index of nonindustrial origin [10].
1983	Xhonga and Valdmanis	Considering extent of erosion in addition to severity [6].
Indices based on Tooth Wear Index (TWI)		
1984	Smith and Knight	Evaluate TSL severity regardless of the etiology [9].
1994	Millward et al.	Adopt TWI criteria to measure erosion TSL in children [17]
2004	Bardsley et al.	Simplified version of TWI to evaluate erosion TSL in children [12].
TSL indices concentrating on treatment needs		
1987	Oil et al.	Tried to provide reliable method to determine the treatment needs for each case [18].
1989	Dahl et al.	Modification of Oilo et al.’s criteria [19].
2000	Larsen et al.	First use of photograph as supplementary tool, with clinical examination [20].
2008	Bartlett et al.	(BEWE) index to provide easy and reproducible diagnostic criteria [13].
2014	Sawai	Simplified indices for cervical lesions [21].
2016	Wetselaar and Lobbezoo	Aimed to provide comprehensive tooth wear evaluation system (TWES) [22].
Use of casts and Three-dimensional methods to classify TSL		
2010	Al-Omiri et al.	Use three-dimensional tools to classify TSL [23].
2015	Lee et al.	Compared the change in tooth volume in a longitudinal comparison of the change in line angles on the occlusal surface of each tooth [15].
2018	Soo-Hyun Kim et al.	New tooth wear index (NTWI) describing methods for quantitative measurement of tooth wear using the area and volume of virtual model cusps [15].

## 2. Materials and Methods

### 2.1. Study Design

The study included an analysis of intraoral photographs and photographs of smiles. These photographs included unidentifiable cropped smile views, as well as anterior, buccal, lingual, and occlusal views. The photographs were varied in terms of severity, aesthetic impact, and age group. The cases were selected equally from the three age categories (12–30, 31–60, and above 60 years) of both male and female gender types.

A further aim was to suggest a treatment strategy for each classification level. Ten clinicians in the prosthodontic secondary care sector (five academic consultants and five specialist registrars), who were teaching or training at the School of Clinical Dentistry at the University of Sheffield and the Charles Clifford Dental Hospital (CCDH) in Sheffield, United Kingdom, agreed to participate to classify the cases. Three hundred photographs in total were collected. These were evaluated and subjected to the inclusion and exclusion criteria.

The inclusion criteria were: all cases with TSL, including erosion, attrition, abrasion, and abfraction on any tooth surface, photographs with high resolution, having the six required views available.

The exclusion criteria were: TSL in children under 12, already restored dentition.

### 2.2. Ethical Considerations

Anonymous, unidentifiable, and untraceable photographs of mild, moderate, and severe tooth wear cases were obtained from previous postgraduate students’ logbooks. The extraoral photographs were cropped to have the smile view only.

### 2.3. Developing the Classification System Tools

**Figure 1:**
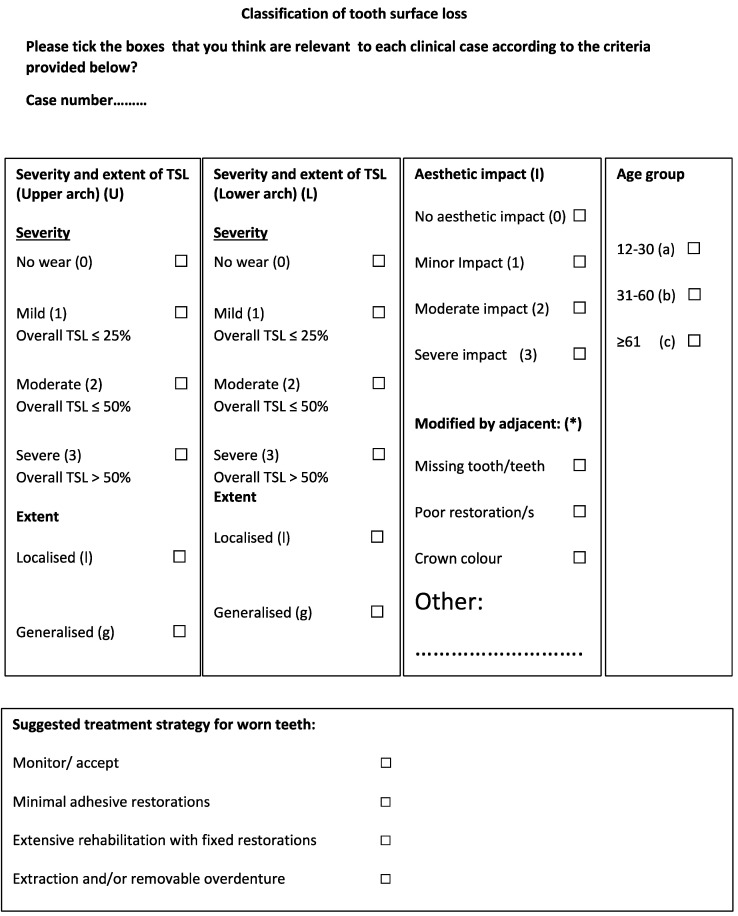
First classification tool in the pilot study.

**Figure 2:**
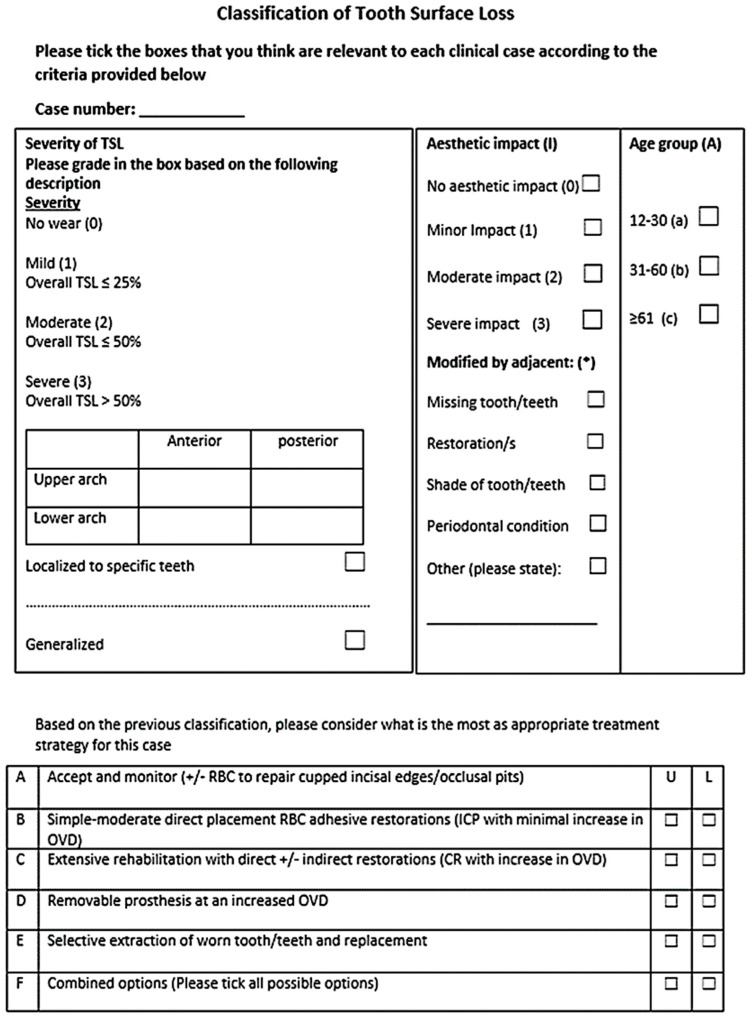
Classification tools for the main study.

Figure 1 shows the first developed classification tool. This form was investigated in a pilot study by 5 academic consultants to identify the source of errors and overcome them. Three main components were considered when categorizing the photos: severity, aesthetic impact, and age. The upper and lower arches were classified separately in the pilot investigation. Figure 2 shows the classification tool for the main study. Both maxillary and mandibular arches were divided into anterior and posterior segments, which could provide a more accurate classification to indicate the level of treatment required.

The aesthetic impact classification (Figure 2) was made based on the following criteria as analyzed from the included photographs:

- No aesthetic impact when the TSL did not cause a noticeable aesthetic impact.
- Minor when there was noticeable TSL on the labial surface or incisal edge, but this did not affect the facial profile and aesthetic appearance.
- Moderate when there was a slight loss of OVD and the facial profile appearance had a noticeable TSL but was not severe.
- Severe aesthetic impact when the TSL was severe and the patient had a severe reduction in OVD with a severely unacceptable appearance due to The TSL itself.

An additional part was added to the aesthetic impact classification, which is a modification or compromising factor and will be scored by (*).

The last part of the classification was the age group (Figure 1).

The treatment options are explained in Figure 1 and were suggested based on acceptable management provided in the literature [2,24,25,26].

### 2.4. Testing the Validity of the Tools

The investigative classification of the form in Figure 2 was carried out by 10 clinicians in the prosthodontic secondary care sector (five academic consultants and five specialist registrars) as described in the study design. Twenty cases were selected randomly for the validity test. Each examiner used the six photographs (smile and intraoral views) for each patient to complete the classification form and provide a suitable management approach. The randomization selection was conducted by numbering the cases and selecting random numbers. The included samples were checked to have all possible classification levels. The cases’ photographs and the classification forms were printed on A4 paper using a SHARP (MX-5141) printer for each examiner.

### 2.5. Analysis

Inter-raters’ agreement and reliability of the results were measured with an intraclass correlation coefficient (ICC) test using SPSS 20 software. The agreement in each case was analyzed by Microsoft Excel software.

## 3. Results

The results of the coefficient correlation analysis between the ten examiners and the lower and upper bounds when the 95% confidence Interval was used are presented in Table 2. The agreement between the examiners showed high reliability when the posterior teeth were scored separately from the anterior teeth.

### 3.1. Severity Classification of Anterior Teeth of Maxillary Arch

Table 3 shows the severity classification entries in the maxillary anterior teeth of 20 cases. Of those, 14 cases had agreement between ≥7 examiners. The most variation in classification was found in Case Numbers 10, 14, 15, and 16. The rate of agreement between the examiners ranged from 0.633 to 0.958, which shows good to very good agreement between them. The only exception was between Examiner 1 and Examiner 6, which showed 0.599 agreement. This is considered moderate according to Altman’s (1991) guidelines for interpreting the strength of agreement.

### 3.2. Severity Classification of Posterior Teeth of Maxillary Arch

Table 3 shows the severity classification entries in the maxillary posterior teeth. Eighteen cases had agreement between seven and nine examiners. The most variation in classification was found in Case Numbers 15 and 18. The lowest agreement was found between Examiner 4 and Examiner 6 (0.63), which is considered good agreement according to Altman’s (1991) guidelines.

### 3.3. Severity Classification of Anterior Teeth of Mandibular Arch

Table 4 shows the severity classification entries in the mandibular anterior sextant of 20 cases. For the severity classification, 14 cases had agreement between ≥7 examiners. The most variation in classification was found in Case Numbers 3, 7, 9, and 11. The rate of agreement between the examiners ranged from 0.65 to 0.98, which shows good to very good agreement between them based on Altman’s (1991) guidelines for interpreting strength of agreement.

### 3.4. Severity Classification of Posterior Teeth of Mandibular Arch

Table 4 shows the severity classification entries in the mandibular posterior sextant. For the severity classification, 100% agreement was found in one case, and 17 cases had agreement between 7 and 9 examiners. The most variation in classification was found in Case Numbers 9, 11, 13, and 17. In two of the cases, only one premolar was present in each side (34, 44) with other posterior teeth extracted; some examiners graded the premolars as a posterior, and others included these two teeth as anterior.

### 3.5. Aesthetic Impact Classification

Table 5 shows the grading entries for the aesthetic impact and the rate of agreement for each case. The agreement between every two examiners ranged between 0.64 and 100%, which is considered good to very good according to Altman (1999) when interpreting the strength of agreement. The lowest agreement was seen in Case Numbers 6 and 11.

### 3.6. Treatment Option Results

The examiners were in agreement that mild cases require just monitoring or simple resin-bonded composite restorations. The variation in the options was apparent when the TSL reached a moderate level. The options given for moderate cases depend mainly on the agreed aim of treatment and whether a confirmative or reorganizing approach should be used. The option of simple direct or indirect restorations can be an option when there is space for restoration. The reorganizing approach is an option when extensive rehabilitation is required at an increased OVD. For severe cases, all examiners agreed with the options that aimed to restore the functionality and aesthetics in CR at an increased OVD.

### 3.7. Providing the Photographic Index Based on Examiners Classification

**Figure 3:**
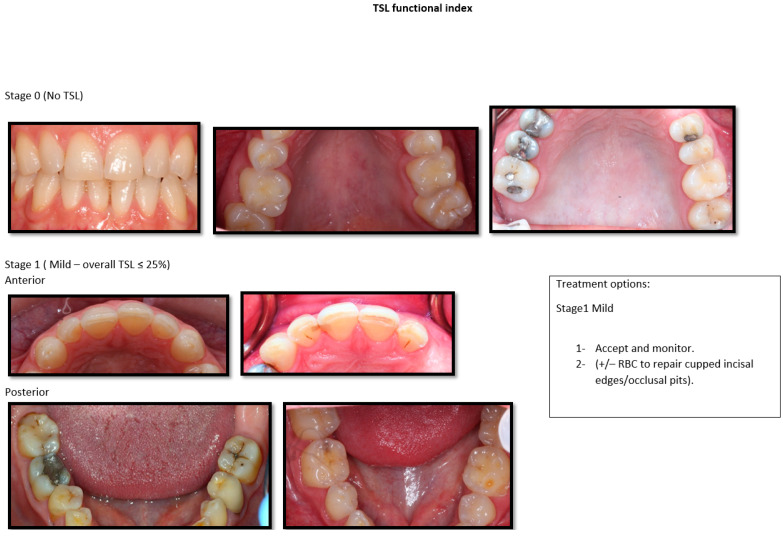
Photographic version of the classification tools with suggested treatment for Stages 0 and 1.

**Figure 4:**
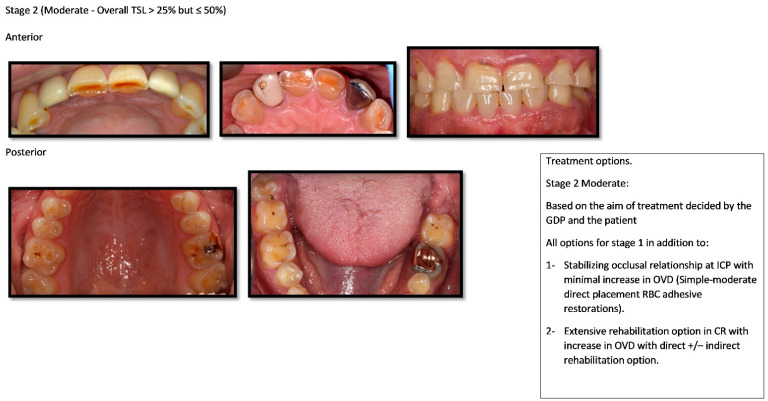
Photographic version of the classification tools with suggested treatment for Stage 2.

**Figure 5:**
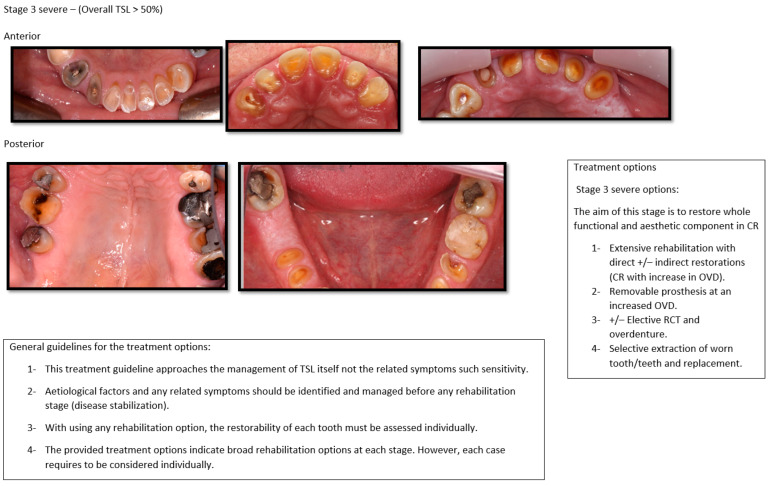
Photographic version of the classification tools with suggested treatment for Stage 3.

As the agreement in the severity classification showed to be a predictable tool, the photographs that were classified were used in providing the photographic version of the classification. Each severity and aesthetic impact classification had four groups, presented in Figure 3, Figure 4 and Figure 5. Mild, moderate, and severe are subgrouped to anterior and posterior, similar to the validated classification tool.

## 4. Discussion

This study is part of a larger project aiming to provide a valid TSL classification using clinical oral photographs that mimic the IOTN system. This study aimed to provide a valid classification tool to classify the clinical photographs for patients having TSL of different severity and aesthetic levels, to provide suggested treatment options for each level, and to use the classified photos to provide a photographic version of the index.

To measure the functional elements, the severity classification system was divided into four stages. These four severity stages are widely used in many indices [9,13]. However, most classifications provide a grade for each tooth or surface, which is time consuming. The Basic Erosive Wear Examination (BEWE) was the first wear index that classified the wear by scoring each sextant in a manner similar to BPE and also considered the management approach [13]. However, it has a low sensitivity in determining the low grades of wear and suggests the management required based on a cumulative score for each sextant [14]. This calculation seems to be difficult to adopt in practice.

The aesthetic impact is considered in this classification system, as it has not been considered in previous indices. The importance of adding aesthetic impact elements to the classification system is to consider the facial appearance and aesthetic considerations during patient assessment and when determining the treatment plan. In some cases, the TSL itself is not the issue compromising the appearance the most. However, the other compromising factors in addition to the TSL can make patients unhappy with their appearance [27]. The reason for adding age to the classification system is to predict the progression of the lesion and to determine the suitable preventive and interventive options based on age.

The management strategies in this study provided a general guide for the possible treatment approaches for each category of tooth wear. The clinician should consider different factors in choosing the treatment, such as the patient’s wishes and concerns and the ability to undertake extensive interventions. The required intervention for each tooth was out of this study’s scope, as the clinician should judge the specific intervention required for each tooth based on the validated restorability indices, which assess the tooth factors, periodontal factors, and endodontic factors. An index, such as the one developed by Dawood and Patel (2017), is considered a good reference to assess the restorability of the teeth considering the mentioned tooth and periodontal and endodontic factors in addition to the strategic value of the tooth [28]. Another index by McDonald and Setchell (2005) provides a guide to the predictability of the teeth based on the remaining tooth structure [29]. For that, it is assumed that the management options should be general, leaving the specific considerations for each tooth to the clinician to assess, based on different restorability indices.

The data analysis was conducted by undertaking Pearson’s coefficient correlation test using SPSS software. This test is a useful method of analysis when quantitative measurements are made on units that are organized based on different groups or examiners, and the strength of the inter-rater measurement is required. In addition, the standard deviation was used to find the cases with high and low agreement. It is beneficial to find the reason for disagreement to modify the tool and provide the most suitable TSL photographs that can be used to represent all levels of TSL in the final version of the index.

The limitation of scoring the full arch by one grade, as identified in the pilot study, was overcome in the main study. It was hypothesized that dividing each arch into anterior and posterior segments in the severity classification would improve the agreement between examiners, which was confirmed by the results. However, some sources of errors can be identified from the second investigation study. The first reason is when only one tooth in the arch has a TSL and the other teeth have been restored. One case had only one premolar with Grade 1 severity, and the other teeth had been restored with no sign of TSL. In this case, some examiners scored the arch as zero, based on the general condition of the site, while others scored the site based on the severest grade, e.g., Case 15 in Table 4. The second reason is that some cases have only two teeth in the posterior segments 34 and 44. Some examiners could have followed the BPE method and scored these two teeth with anterior teeth in one score, while other examiners may have provided a different classification, as the premolars are considered from the posterior teeth. These two reasons can be avoided by providing clear instructions for the classification. Other sources of error, such as scoring errors, cannot be excluded.

The aesthetic impact on TSL had good agreement between examiners. The variation between examiners can be related to the subjective nature of the evaluation of the aesthetic impact between them. Furthermore, no strict criteria were given to differentiate between a mild, moderate, or severe impact. The positive finding is that all examiners agreed that some compromising factors can compromise the aesthetic impact. It is useful for the clinician to be able to judge the level of aesthetic impact using the dental aesthetic guidelines and the golden proportion between the teeth to determine the intervention level that can be provided to improve the patient’s appearance. When applying this classification clinically, patients’ expectations, opinions, and concerns about the aesthetic should also be considered.

Regarding the suggested treatment, there was agreement that cases with mild TSL should be monitored or apply direct restoration conforming to the occlusion. This is confirmed by the results of different studies [20,30,31]. Moderate and severe cases seem to have been more complex to plan, as was expected. However, it was agreed that all cases with generalized moderate to severe TSL should be rehabilitated at the centric relation to an increased OVD. The option of rehabilitation can vary from fixed or removable options and can include a surgical intervention using crown lengthening surgery. The aim of treatment at the moderate level can determine the required amount of intervention. In some cases where the function is not compromised, the conforming at ICP with minimal intervention can satisfy the patient and speed up the treatment time. In severe cases, the treatment should aim to restore the functionality of the teeth at CR. Then, more extensive options are inevitable at this level, which agrees with the study conducted by Chu et al. (2017) [25].

Although this preliminary study showed high reliability between ten examiners, further studies can be conducted with more strict classification criteria. It will be beneficial to include general dentists in the following study, which can help to assess the reliability of the classification tools and the supporting photographic version between general dentists.

## 5. Conclusions

The use of photographs in this study is considered to be an appropriate tool for classifying the severity of TSL. Dividing the mouth into anterior and posterior sites in each arch provided a high level of agreement in the severity classification. The highest agreement between examiners was found in the mandibular anterior wear classification, while the lowest agreement was found in the mandibular posterior tooth wear classification. The use of photographs in the aesthetic impact classification provided a high level of agreement between examiners in most cases. However, patient concerns should be considered in practice in addition to the clinician’s judgment. Moreover, the quality of photographs and the appearance of all surfaces should be considered when photographs are used to assess tooth wear. In terms of management options, the examiners agreed on the required intervention at each level. This preliminary study proved the agreed approach of monitoring mild cases, simple restoration in mild cases, and advanced treatment plans for severe cases. However, in this study, the aesthetic consideration as an additional indicator can influence the treatment option. Further investigation for the photographic version is required, and some patient factors that affect the restorability of the teeth need to be considered.

## Figures and Tables

**Table 2:** Interexaminer correlation coefficient from the main study.

		95% Confidence Interval	
	Intraclass Correlation	Lower Bound	Upper Bound
Maxillary anterior arch	0.805	0.70	0.91
Maxillary posterior arch	0.81	0.71	0.91
Mandibular anterior arch	0.845	0.76	0.93
Mandibular posterior arch	0.76	0.64	0.88
Aesthetic impact	0.825	0.73	0.92

**Table 3:** Range of agreement between examiners in each case (upper arch).

**Number of samples**		Number of Examiners Scored Grades 0–3 for Each Sample							
		Maxillary anterior				Maxillary posterior			
		0	1	2	3	0	1	2	3
	1			3	7			1	9
	2		10			3	7		
	3			1	9			8	2
	4	2	8				1	9	
	5		2	1	7			8	2
	6			2	8		7	3	
	7			3	7	1	9		
	8		1	2	7		3	6	1
	9				10	2	7	1	
	10			5	5	1	9		
	11			1	9			3	7
	12	7	3				7	3	
	13			2	8	9	1		
	14		2	6	2		8	2	
	15		2	6	2	5	5		
	16		5	5		1	9		
	17				10	7	3		
	18				10	4	6		
	19		8	2		7	3		
	20			3	7	1	9		

**Table 4:** Range of agreement between examiners in each case (lower arch).

**Number of samples**		Number of Examiners Scored Grades 0–3 for Each Sample							
		Mandibular anterior				Mandibular posterior			
		0	1	2	3	0	1	2	3
	1			1	9			8	2
	2	1	9			8	2		
	3		1	6	3			8	2
	4	9	1					2	8
	5		8	2			7	3	
	6		10			1	9		
	7		1	7	2	2	8		
	8				10		3	7	
	9		1	5	4	2	1	7	
	10	3	7				8	2	
	11		1	3	6			4	6
	12	9	1				7	3	
	13		6	4			5	5	
	14				10				10
	15		8	2		3	7		
	16		4	6			8	2	
	17		1	8	1	3	6	1	
	18			4	6	1	9		
	19		9	1		2	8		
	20			1	9	1	9		

**Table 5:** Range of agreement between examiners in each case (aesthetic impact).

**Number of samples**		Number of Examiners Scored Grades 0–3 for Each Sample			
		0	1	2	3
	1			8	2
	2		10		
	3		1	9	
	4	8	2		
	5		1	9	
	6			6	4
	7		1	1	8
	8			1	9
	9			1	9
	10		1	8	1
	11			5	5
	12	8	2		
	13			9	1
	14		3	7	
	15		1	9	
	16	8	2		
	17				10
	18			3	7
	19		1	9	
	20		1	1	8

## Data Availability

Not Applicable.

## Abbreviations

TSL	Tooth Surface Loss
OVD	Occlusal Vertical Dimension
IOTN	Index for Orthodontic Treatment Needs
TWI	Tooth Wear Index
BEWE	Basic Erosive Wear Examination
CAD-CAM	Computer Aided Design—Computer Aided Manufacturing
CCDH	Charles Clifford dental hospital
BPE	Basic Periodontal Examination

## References

[1] Cunha-Cruz J., Pashova H., Packard J.D., Zhou L., Hilton T.J. (2010). Tooth wear: Prevalence and associated factors in general practice patients. Community Dent. Oral Epidemiol..

[2] Ahmed K.E., Murbay S. (2016). Survival rates of anterior composites in managing tooth wear: Systematic review. J. Oral Rehabil..

[3] Bartlett D.W., Lussi A., West N.X., Bouchard P., Sanz M., Bourgeois D. (2013). Prevalence of tooth wear on buccal and lingual surfaces and possible risk factors in young European adults. J. Dent..

[4] Li M.H.M., Bernabé E. (2016). Tooth wear and quality of life among adults in the United Kingdom. J. Dent..

[5] Todic J., Mitic A., Lazic D., Radosavljevic R., Staletovic M. (2017). Effects of bruxism on the maximum bite force. Vojnosanit. Pregl..

[6] Geographic Comparisons of the Incidence of Dental Erosion: A Two Centre Study. https://onlinelibrary.wiley.com/doi/abs/10.1111/j.1365-2842.1983.tb00121.x.

[7] Vanuspong W., Eisenburger M., Addy M. (2002). Cervical tooth wear and sensitivity: Erosion, softening and rehardening of dentine; effects of pH, time and ultrasonication. J. Clin. Periodontol..

[8] Katsoulis J., Nikitovic S.G., Spreng S., Neuhaus K., Mericske-Stern R. (2011). Prosthetic rehabilitation and treatment outcome of partially edentulous patients with severe tooth wear: 3-Years results. J. Dent..

[9] Bardsley P.F. (2008). The evolution of tooth wear indices. Clin. Oral Investig..

[10] Eccles J.D. (1979). Dental erosion of nonindustrial origin. A clinical survey and classification. J. Prosthet. Dent..

[11] Ganss C., Lussi A. (2008). Current erosion indices—Flawed or valid?. Clin. Oral Investig..

[12] Bardsley P.F., Taylor S., Milosevic A. (2004). Epidemiological studies of tooth wear and dental erosion in 14-year-old children in North West England. Part 1: The relationship with water fluoridation and social deprivation. Br. Dent. J..

[13] Bartlett D., Ganss C., Lussi A. (2008). Basic Erosive Wear Examination (BEWE): A new scoring system for scientific and clinical needs. Clin. Oral Investig..

[14] Bartlett D. (2012). Summary of: Evaluation of the basic erosive wear examination (BEWE) for use in general dental practice. Br. Dent. J..

[15] Kim S.H., Park Y.S., Kim M.K., Kim S., Lee S.P. (2018). Methods for quantitative measurement of tooth wear using the area and volume of virtual model cusps. J. Periodontal. Implant. Sci..

[16] Brook P.H., Shaw W.C. (1989). The development of an index of orthodontic treatment priority. Eur. J. Orthod..

[17] Millward A., Shaw L., Smith A.J., Rippin J.W., Harrington E. (1994). The distribution and severity of tooth wear and the relationship between erosion and dietary constituents in a group of children. Int. J. Paediatr. Dent..

[18] Oilo G., Dahl B.L., Hatle G., Gad A.L. (1987). An index for evaluating wear of teeth. Acta Odontol. Scand..

[19] Dahl B.L., Oilo G., Andersen A., Bruaset O. (1989). The suitability of a new index for the evaluation of dental wear. Acta Odontol. Scand..

[20] Larsen I.B., Westergaard J., Stoltze K., Larsen A.I., Gyntelberg F., Holmstrup P. (2000). A clinical index for evaluating and monitoring dental erosion. Community Dent. Oral Epidemiol..

[21] Sawai M. (2014). An easy classification for dental cervical abrasions. Dent. Hypotheses.

[22] Wetselaar P., Lobbezoo F. (2016). The tooth wear evaluation system: A modular clinical guideline for the diagnosis and management planning of worn dentitions. J. Oral Rehabil..

[23] AL-Omiri M.K., Harb R., Hammad O.A.A., Lamey P.J., Lynch E., Clifford T.J. (2010). Quantification of tooth wear: Conventional vs new method using toolmakers microscope and a three-dimensional measuring technique. J. Dent..

[24] Satterthwaite J.D. (2017). Tooth surface loss: Tools and tips for management. Dent. Update.

[25] Chu F.C.S., Chow T.W., Smales R.J., Newsome P.R.H., Siu A.S.C. (2017). Restorative Management of the Worn Dentition: 4. Generalized Toothwear. Dent. Update.

[26] Abduo J., Lyons K. (2012). Clinical considerations for increasing occlusal vertical dimension: A review. Aust. Dent. J..

[27] Lee S., Nam S., Lee S. (2015). Evaluation of the effectiveness of the new tooth wear measurement parameters. Anat. Cell Biol..

[28] Dawood A., Patel S. (2017). The Dental Practicality Index-assessing the restorability of teeth. Br. Dent. J..

[29] McDonald A., Setchell D. (2005). Developing a tooth restorability index. Dent. Update.

[30] West N.X., Joiner A. (2014). Enamel mineral loss. J. Dent..

[31] Al-Khayatt A.S., Ray-Chaudhuri A., Poyser N.J., Briggs P.F.A., Porter R.W.J., Kelleher M.G.D., Eliyas S. (2013). Direct composite restorations for the worn mandibular anterior dentition: A 7-year follow-up of a prospective randomised controlled split-mouth clinical trial. J. Oral Rehabil..

